# Enhancing generalizability and performance in drug–target interaction identification by integrating pharmacophore and pre-trained models

**DOI:** 10.1093/bioinformatics/btae240

**Published:** 2024-06-28

**Authors:** Zuolong Zhang, Xin He, Dazhi Long, Gang Luo, Shengbo Chen

**Affiliations:** School of Software, Henan University, Kaifeng, Henan Province 475000, China; School of Software, Henan University, Kaifeng, Henan Province 475000, China; Henan International Joint Laboratory of Intelligent Network Theory and Key Technology, Henan University, Kaifeng, Henan Province 475000, China; Department of Urology, Ji’an Third People’s Hospital, Ji’an, Jiangxi Province 343000, China; School of Mathematics and Computer Science, Nanchang University, Nanchang, Jiangxi Province 330031, China; Henan Engineering Research Center of Intelligent Technology and Application, Henan University, Kaifeng, Henan Province 475000, China

## Abstract

**Motivation:**

In drug discovery, it is crucial to assess the drug–target binding affinity (DTA). Although molecular docking is widely used, computational efficiency limits its application in large-scale virtual screening. Deep learning-based methods learn virtual scoring functions from labeled datasets and can quickly predict affinity. However, there are three limitations. First, existing methods only consider the atom-bond graph or one-dimensional sequence representations of compounds, ignoring the information about functional groups (pharmacophores) with specific biological activities. Second, relying on limited labeled datasets fails to learn comprehensive embedding representations of compounds and proteins, resulting in poor generalization performance in complex scenarios. Third, existing feature fusion methods cannot adequately capture contextual interaction information.

**Results:**

Therefore, we propose a novel DTA prediction method named HeteroDTA. Specifically, a multi-view compound feature extraction module is constructed to model the atom–bond graph and pharmacophore graph. The residue concat graph and protein sequence are also utilized to model protein structure and function. Moreover, to enhance the generalization capability and reduce the dependence on task-specific labeled data, pre-trained models are utilized to initialize the atomic features of the compounds and the embedding representations of the protein sequence. A context-aware nonlinear feature fusion method is also proposed to learn interaction patterns between compounds and proteins. Experimental results on public benchmark datasets show that HeteroDTA significantly outperforms existing methods. In addition, HeteroDTA shows excellent generalization performance in cold-start experiments and superiority in the representation learning ability of drug–target pairs. Finally, the effectiveness of HeteroDTA is demonstrated in a real-world drug discovery study.

**Availability and implementation:**

The source code and data are available at https://github.com/daydayupzzl/HeteroDTA.

## 1 Introduction

The screening of potential drug candidates from the compound library is a crucial step in drug development. The assessment of drug–target binding affinity (DTA) is a critical task as it determines the strength of binding between a compound and its target. Traditional screening methods fall into two types. Wet experiments require assays and validation in biological systems, which are costly and time-consuming. Although molecular docking can use computers to simulate the binding process through space and energy matching, it is equally time-consuming and laborious in large-scale virtual screening (Li *et al.*, 2022). Therefore, there is an urgent need for the development of efficient and accurate computational methods to identify DTA.

With the increasing data in drug development, high-throughput computational methods for DTA prediction have been proposed, which can be divided into two types: machine learning (ML)-based methods and deep learning (DL)-based methods. Early studies used ML methods to predict DTA, including random forest and logistic regression. For example, KronRLS employs a kernel-based approach to generate compound descriptors (Pahikkala *et al.*, 2014). SimBoost uses collaborative filtering technology to calculate the affinity similarity between compounds and targets, which is used as the feature vector for DTA prediction (He *et al.*, 2017). Compared to ML methods, DL methods can handle complex and large-scale data and extract features automatically, which have achieved remarkable success in bioinformatics (Sadybekov and Katritch, 2023). For example, DeepDTA utilizes convolutional neural networks (CNN) to learn features of SMILES strings of compounds and amino acid sequences of proteins (Öztürk *et al.*, 2018). To utilize the topological information of the compounds, GraphDTA constructs a two-dimensional topological graph with atoms as nodes and chemical bonds as edges and then uses graph neural networks (GNN) to learn structural information (Nguyen *et al.*, 2020). In addition, to capture the spatial structure information of proteins, WGNN-DTA and GSAML-DTA employ protein structure prediction models to predict residue concat graphs, and then GNN is used to learn the structural features of proteins (Jiang *et al.*, 2022; Liao *et al.*, 2022).

Although prior studies have made significant progress, there are still several notable limitations in the DTA task. First, existing methods primarily rely on SMILES strings or atom–bond graphs to analyze compounds, overlooking the crucial role of pharmacophores, which are specific fragments of chemical structures with distinct pharmacological activities that significantly influence the selectivity and potency of compounds (Choudhury and Sastry, 2019). Therefore, the role of pharmacophores on compound properties needs to be considered in the encoding process. Second, existing methods are based on small DTA datasets, such as Davis (Davis *et al.*, 2011) and KIBA (Tang *et al.*, 2014). These datasets have limited coverage of compounds and proteins compared to large databases (e.g. PubChem and UniProt). Thus, there are limitations in learning comprehensive feature representations that make it difficult to reliably predict the affinity of unknown compounds or proteins. However, how to improve the performance and generalization ability of DTA prediction methods on limited DTA datasets has not been sufficiently investigated. Finally, existing methods often employ non-context-aware linear strategies for feature fusion, such as direct concatenation, which cannot comprehensively capture the complex contextual interaction information between compounds and targets. Consequently, a nonlinear feature fusion mechanism that effectively captures the context-awareness of these complex interactions is needed for improving the accuracy and reliability of DTA prediction.

To address the aforementioned limitations, a deep learning-based DTA prediction method named HeteroDTA is proposed in this article. Specifically, for compounds, a multi-view compound feature extraction module is designed that not only contains atom–bond graphs but also identifies and models pharmacophores. The hybrid approach can extract key features from functional substructures of the compounds and learn structural features comprehensively. For proteins, both residue concat graphs and protein sequences are used to capture structural and functional features. In addition, to enhance the generalization ability of the model on limited DTA datasets, two advanced pre-trained representation learning models are introduced. For compounds, to enhance the ability to extract feature representations of unknown compounds, a geometrically enhanced molecular representation learning model (GEM (Fang *et al.*, 2022)) is used, which is pre-trained on a large library of organic small molecule compounds. For proteins, to extract structural and hidden evolutionary information directly from the sequence, a transformer-based protein pre-trained model (ESM-1b (Rives *et al.*, 2021)) is used, which is pre-trained on large-scale sequence databases. In the feature fusion block, to overcome the limitations of traditional methods in capturing complex contextual interaction information, a context-aware nonlinear feature fusion mechanism is proposed, which is different from the direct concat fusion approach of the existing methods and can achieve more detailed combination and interaction between compound features and target features. Experiments on several publicly available datasets show that HeteroDTA outperforms existing methods on all metrics. In particular, HeteroDTA exhibits significant generalization ability in various complex cold-start scenarios. Moreover, cluster analysis of drug–target pairs further confirms the power of HeteroDTA in representation learning. Finally, in a case study focused on the core target of severe acute respiratory syndrome coronavirus 2 (SARS-CoV-2), HeteroDTA is used to perform an efficient virtual screening, demonstrating its superior ability to discover potential candidates in a real-world drug discovery scenario.

## 2 Methods

### 2.1 Overview of HeteroDTA

**Figure 1.**
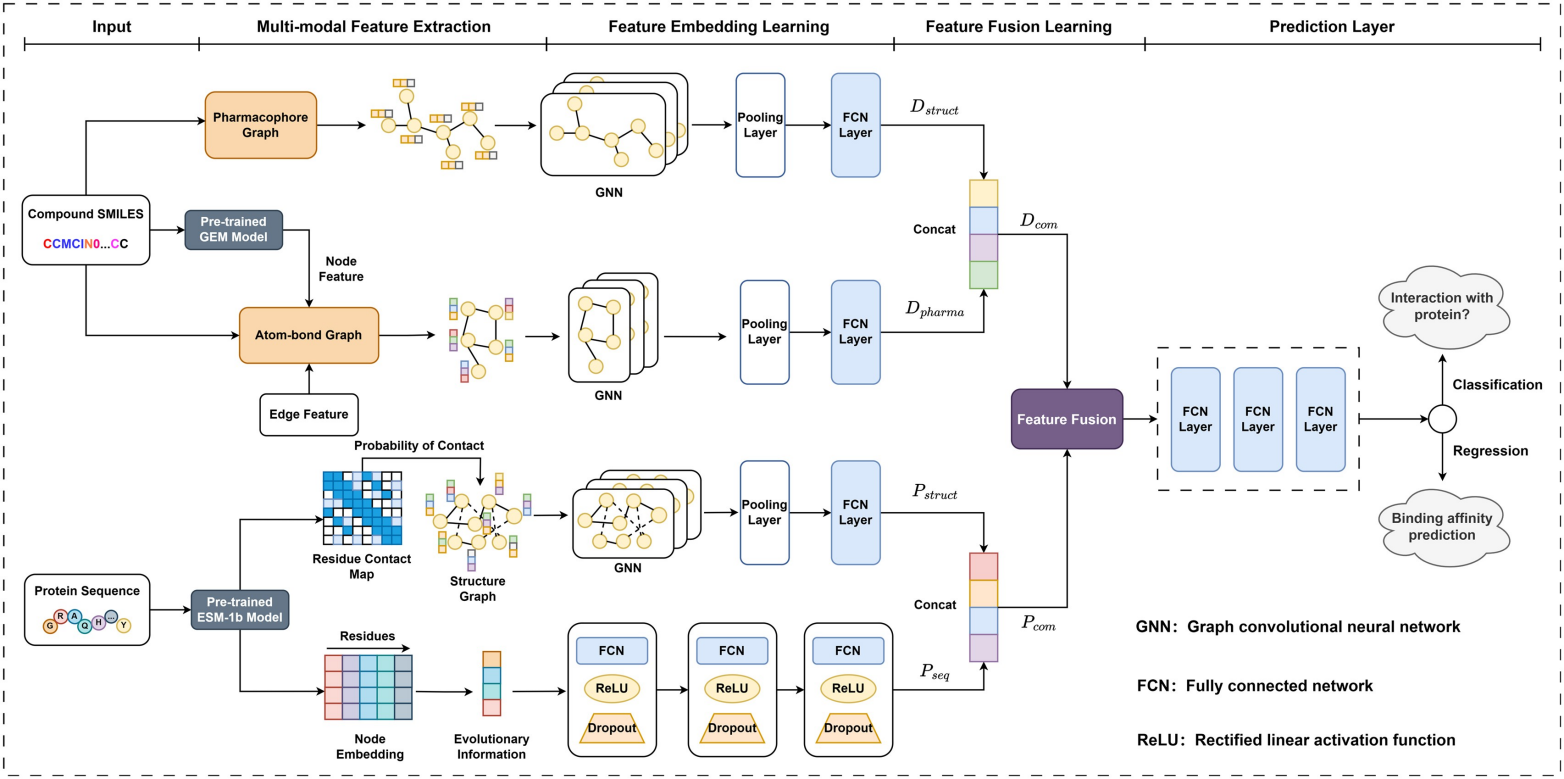
Overarchitecture of the HeteroDTA.

The model architecture of HeteroDTA is shown in Figure 1. In the feature extraction layer, for compounds, atom–bond graphs and pharmacophores are constructed respectively, and features of chemical bonds are collected to indicate bond types and chirality information. The atom embeddings in the atom–bond graph are initialized using the pre-trained model GEM. For proteins, the protein sequence is fed into the pre-trained model ESM-1b to predict the residue concat graph and residue embedding representation respectively, and then the weighted residue concat graph is constructed, where the weights of edges refer to the concat probabilities between residues. In the feature embedding layer, GNN is used to aggregate the features and edge features of adjacent nodes, and the features of nodes are updated iteratively. The graph-level embedding representation is obtained through the max pooling layer. The implicit evolutionary information in protein sequence is learned using multilayer perceptron networks. In the feature fusion layer, a context-aware nonlinear feature fusion method is designed, which can capture the binding pattern and generate the fused feature representation. In the prediction layer, multilayer perceptron networks are used to learn the fusion feature representation to predict the binding affinity between the compound and target or whether there will be an interaction between them (DPI).

### 2.2 GNN block

In the feature extraction module, GNN is used to learn the feature representations of the pharmacophore graph, atom–bond graph, and residue concat graph. GNN can comprehensively consider the characteristics of the node itself and the information of its neighbors, to represent the features of compounds and proteins more comprehensively. Two variants of GNN are used in this study: graph convolutional neural network (GCN) and graph attention neural network (GAT) (Kipf and Welling, 2017; Veličković *et al.*, 2018).

GCN uses the connection relationship between nodes for information transfer, and updates and aggregates node features by convolution operation of adjacency matrix. The propagation formula is as follows:

(1) $$H^{\left(l+1\right)}=\sigma \left({\tilde{D}}^{–\frac{1}{2}}\tilde{A}{\tilde{D}}^{–\frac{1}{2}}H^{\left(l\right)}W^{\left(l\right)}\right),$$

where $\tilde{A}=A+I_{N}$ denotes the adjacency matrix with self-loops. *I_N_* is the identity matrix used to add self-loops at each node to take into account the characteristics of each node itself. $\tilde{D}$ is the degree matrix of the graph. $H^{\left(l\right)}$ represents the feature matrix of the *l* layer. $H^{\left(l+1\right)}$ is the output feature matrix after graph convolution. *σ* is the ReLU activation function. $W^{\left(l\right)}$ is the learnable weight matrix of the *l* layer.

Different from GCN, GAT uses a learnable attention mechanism to aggregate the features between nodes, thereby capturing the importance differences between different nodes. Specifically, for each node, its feature vector is summed with the feature vectors of neighboring nodes and multiplied by the learnable attention coefficient. These coefficients can adaptively learn how much each neighbor node contributes to the current node. Suppose there are two nodes, denoted as *h_i_* and *h_j_*. Firstly, through a learnable linear transformation matrix *W*, the embedded representations of nodes are projected into higher-level features to improve the expressivity, and then concatenation is performed. Next, the feedforward neural network ${\vec{a}}^{\;T}$ is a single-layer perceptron network containing trainable parameters designed to project connected high-dimensional features to a real number and then activate it using the LeakyReLU activation function. The attention coefficient *a_ij_* is used to indicate the importance of node *h_i_* to node *h_j_* and is calculated as follows:

(2) $$e_{ij}=\text{LeakyReLU}\left({\vec{a}}^{\;T}\left[W{\vec{h}}_{i}||W{\vec{h}}_{j}\right]\right)$$

(3) $$a_{ij}=\text{softmax}(e_{ij})=\frac{\;\text{exp}(e_{ij})}{\sum_{k\in N_{i}}\;\text{exp}\;(e_{ik})},$$

where *N_i_* denotes all neighbors of the *i*th node and || denotes the concatenation. After obtaining the attention coefficients, the output features $h_{i}^{\prime }$ of node *i* are obtained by weighted summation of neighboring nodes:

(4) $$h_{i}^{\prime }=\sigma \left(\sum_{j\in N_{i}}a_{ij}W{\vec{h}}_{j}\right).$$

To enhance the representation ability and stability of the model, a multi-head attention mechanism is introduced here, which connects several different heads to form the output features $\hat{h}\prime_{i}$, as follows:

(5) $$\hat{h}\prime_{i}={∥}_{k=1}^{K}\sigma \left(\sum_{j\in N_{i}}\alpha_{ij}^{k}W^{k}{\vec{h}}_{j}\right),$$

where $\hat{h}\prime_{i}$ consists of *K* different heads connected to improve the representation power of the GNN model.

### 2.3 Pre-trained models

In DTA tasks, capturing sufficient compound and protein features is challenging due to the complexity of the data and limitations. Therefore, two advanced pre-trained models are used in this study: GEM and ESM-1b. As shown in Supplementary Figure S1, for compounds, RDKit (Landrum, 2014) is used to extract the 3D conformational information, including atom–bond graphs and bond–angle graphs. The above two types of graph information are then fed into the GEM model to obtain a 32-dimensional embedded feature representation of the individual atoms in the compound. For proteins, amino acid sequences are input into the ESM-1b model, which can predict interaction probability between residues as well as the embedding representation of each residue directly from the sequence. Subsequently, the residue embedding representation is averaged according to the residue dimension to obtain a 1280-dimensional feature representation of the protein sequence.

### 2.4 Compound encoding block

In this section, pharmacophore graphs and atom–bond graphs are constructed to capture structural and functional information about compounds from different perspectives. The pharmacophore graph represents functional groups as nodes and their connections as edges, which can identify chemical features within a compound. On the other hand, the atom–bond graph represents atoms as nodes and chemical bonds as edges, which can capture the microstructural information of a compound. Through the combination of these two graphs, the macroscopic and microscopic features of compounds can be captured simultaneously, providing a comprehensive and accurate description method for feature extraction of compounds.

#### 2.4.1 Pharmacophore graph encoding

**Figure 2.**
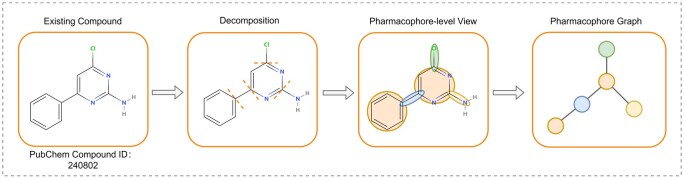
Overview of pharmacophore graph construction. The process consists of three steps: (1) Decomposition of the given compound graph using predefined strategies. (2) Identification and extraction of pharmacophore nodes, with connections established between them. (3) Integration of these elements to construct a complete pharmacophore graph.

Pharmacophores are chemical groups in a compound that have an influence on chemical and physical properties and play a crucial role in drug development (Maslehat *et al.*, 2018). For example, heterocyclic structures are present in over 85% of FDA-approved drugs and significantly influence the physical properties and biological activities of drugs (Hossain *et al.*, 2023). However, most current DTA prediction methods often rely on the atom–bond graph, overlooking critical pharmacophore information such as heterocyclic structures. This oversight can lead to the loss of key features in drug–target interactions. Therefore, we propose a pharmacophore graph encoding method. As shown in Figure 2, all cyclic structures within compounds are initially labeled as nodes, and individual chemical bonds in non-cyclic structures and the connected atoms are considered as nodes as well. Edges are subsequently established between neighboring nodes. Detailed chemical and physical properties of all the atoms and chemical bonds contained within each node are then extracted to initialize node features (refer to Supplementary Table S1 for more details). Finally, we use GAT as the core feature extraction module, which effectively identifies and learns critical features within pharmacophore graphs.

#### 2.4.2 Atom–bond graph encoding

In this study, we utilize the SMILES strings of compounds as input and process them using RDKit to generate atom–bond graphs, representing each atom as a node and chemical bonds as edges. The pre-trained GEM model is introduced to initialize the feature representation of each node in the atom–bond graph. Combining the GEM model with the atom–bond graph aims to enhance the model’s understanding of the compound structure and improve its generalization performance. To capture the complexity of compounds, we added bond type and chirality information as edge attributes (see Supplementary Table S2 for details). Subsequently, we constructed a compound feature embedding module based on a multilayer GAT network. By aggregating the node features in the multilayer network, our model can capture atom-level structural feature information within compounds.

### 2.5 Protein encoding block

In this study, we investigate proteins from two dimensions: conformation and sequence. To obtain conformational information, we use the protein structure prediction model ESM-1b to infer the residue contact information of proteins end-to-end, and then construct a weighted residue contact graph. Specifically, each residue is treated as a node in the graph, and when the contact probability between residues exceeds 0.5, it is considered to be a stable contact, and then this relationship is established as a connecting edge; if it is below this threshold, it is considered to be no contact. In addition, we use the contact probability as the weight of the connecting edges, reflecting the relative strengths of the different contacts, thus capturing the protein conformation comprehensively. In addition, a set of residual descriptors is used to describe the initial features of each residue (see Supplementary Table S3 for details). Finally, we combine multilayer GCN with GAT to construct a conformational learning module that aggregates node and edge features. To capture the evolutionary information in sequences, the embedding of individual residues is extracted using the ESM-1b model, and then the sequence-level embedding is obtained by averaging the residue-level embeddings. At the feature embedding layer, multilayer perceptron networks are used to learn the evolutionary information in the sequence-level embedding.

### 2.6 Interaction learning

**Figure 3.**
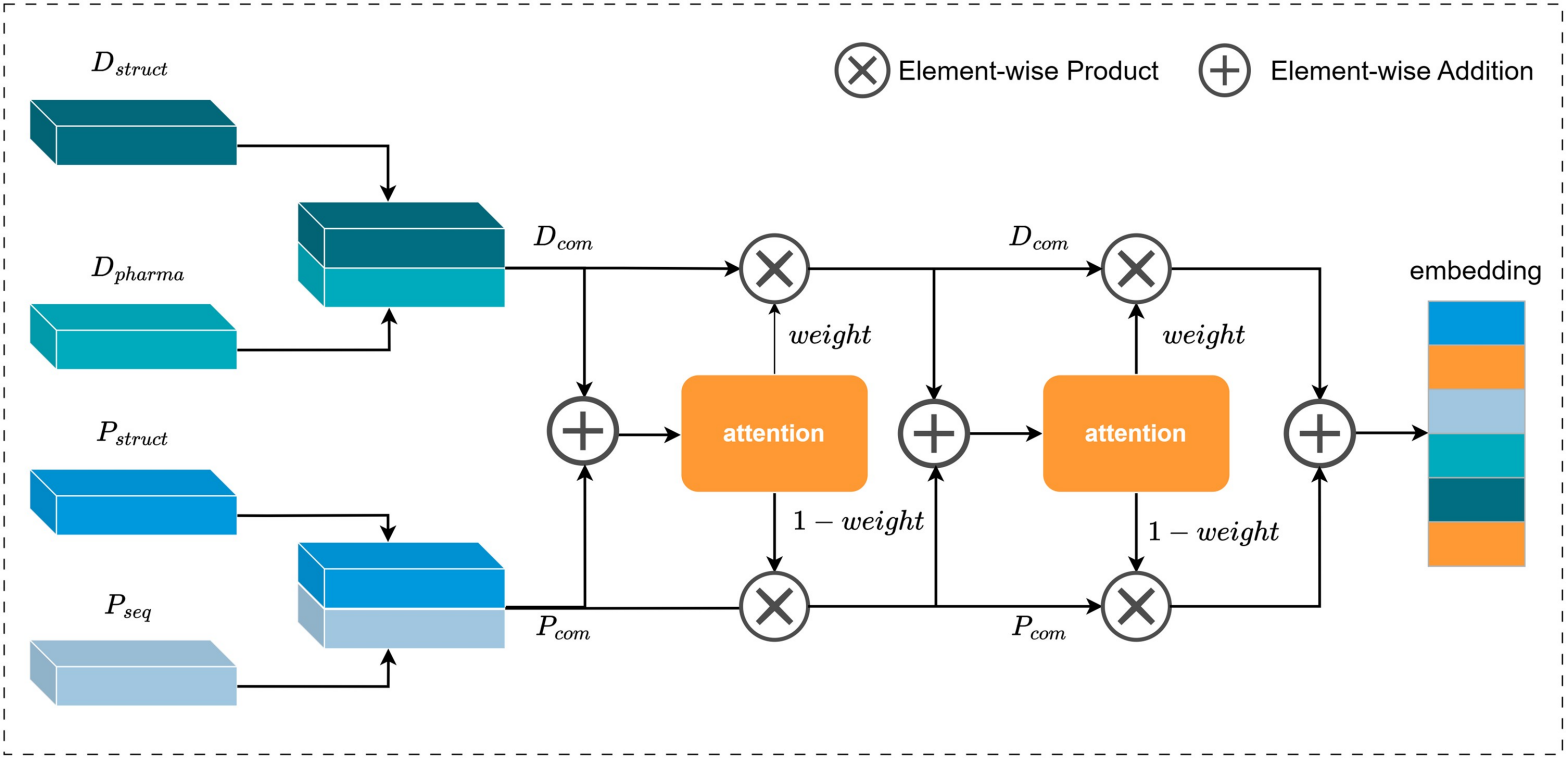
Illustration of context-aware nonlinear feature fusion mechanism.

Inspired by Dai *et al.* (2021), a context-aware nonlinear feature fusion mechanism is proposed to better capture the interaction patterns between drugs and targets, as shown in Figure 3. This mechanism, by introducing nonlinear activation functions and learnable parameterized mappings, dynamically adjusts the importance of features based on the context information of input features. It effectively simulates the interaction between drugs and targets, significantly enhancing the performance of models in DTA prediction tasks. Note that the output of the last layer of each graph feature embedding learning block in HeteroDTA is fed into a max pooling layer, which can ignore the differences in node sets and number of nodes to obtain a graph-level embedding representation.

Firstly, atom–bond graph features *D_struct_* and pharmacophore graph features *D_pharma_* are extracted from compounds, while structural features *P_struct_* and sequence-level evolutionary feature *P_seq_* are extracted from proteins. These features are concatenated into compound embedding $D_{\mathrm{com}}=D_{\mathrm{struct}}||D_{\mathrm{pharma}}$ and protein embedding $P_{\mathrm{com}}=P_{\mathrm{struct}}||P_{\mathrm{seq}}$, respectively. Next, the formula for calculating attention weights is as follows:

(6) $$\text{attention}=W_{2}\left(\text{ReLU}\left(W_{1}x+b_{1}\right)\right)+b_{2},$$

where *W*_1_ and *W*_2_ are trainable weights, and *b*_1_ and *b*_2_ are bias terms.

Second, *D_com_* and *P_com_* are input into the attention network to obtain the attention coefficient weight, which is calculated as follows:

(7) $$\text{weight}=\text{Sigmoid}(\text{attention}(D_{\mathrm{com}}+P_{\mathrm{com}})).$$

Finally, *D_com_* and *P_com_* are weighted summed using attention coefficients weight to obtain the fused feature representation embedding, as described by the following formula, where ⊙ represents element-wise multiplication:

(8) $$\text{embedding}=D_{\mathrm{com}}⊙\text{weight}+P_{\mathrm{com}}⊙(1-\text{weight}).$$

### 2.7 Prediction layer

The prediction layer employs multilayer perceptron networks using the ReLU activation function and Dropout regularization. For the DPI prediction task, a Sigmoid activation function is added at the last layer to output the probability of their interactions in the range of 0 to 1:

(9) $$\hat{Y}=\text{Sigmoid}(\text{FCN}(\text{embedding})).$$

## 3 Experiments

### 3.1 Dataset preparation

To test the performance of the HeteroDTA model, four widely used public datasets are chosen (see Table 1). The KIBA and Davis datasets are used for the DTA task, while the Human and *Caenorhabditis elegans* datasets are used for the DPI task (Liu *et al.*, 2015). These datasets contain information on multiple drugs and targets, as well as binding affinity data between them, and are widely used to evaluate different DPI and DTA prediction methods. For a detailed data distribution of these benchmark datasets, please see Supplementary Figure S2.

**Table 1.** Details of benchmark dataset.

Dataset	Compounds	Targets	Samples
KIBA	2111	229	118 254
Davis	68	442	30 056
Human	2573	1764	5837
*C.elegans*	1733	1735	6995

### 3.2 Baseline models and evaluation metrics

In this study, HeteroDTA is compared with the following benchmark models focusing on different aspects: SimBoost, DeepDTA, DeepCDA (Abbasi *et al.*, 2020), GraphDTA, WGNN-DTA, and GSAML-DTA. A range of evaluation metrics are used to assess the accuracy and reliability of these models in different tasks. For the regression task, the mean square error (MSE), confidence interval (CI), Pearson’s correlation coefficient (Pearson), Spearman’s rank correlation coefficient (Spearman), and the $r_{m}^{2}$ indicator are used. $r_{m}^{2}$ is a special regression task evaluation metric proposed in DeepDTA to measure the similarity between predicted and actual values. For the classification task, four metrics are used: AUC, Precision, Recall, and F1-score. For the clustering task, three indicators are used to comprehensively evaluate the clustering effect, namely the Silhouette Coefficient (SC), Calinski–Harabasz Index (CHI), and Davies–Bouldin Index (DBI). The SC index is used to measure the similarity within a data point and its cluster and the dissimilarity between different clusters. CHI aims to measure the difference between the variance within a cluster and the variance between clusters to reflect the closeness and separation of clusters. A higher CHI value indicates a higher separation between clusters and a better clustering effect. DBI is designed to measure the similarity between different clusters versus the closeness within clusters. A lower DBI value indicates high intra-cluster tightness and low similarity between different clusters.

### 3.3 Training protocol and hyperparameters

The HeteroDTA model designed in this study is suitable for two tasks: DTA prediction and DPI prediction. The model is trained using the mean square error loss function and cross-entropy loss function, respectively. To ensure comparability of study results, the same dataset splitting method and training testing strategy as for all benchmark models are used. Specifically, for the DTA task, the benchmark dataset is divided into six folds, and the model is first iteratively trained on five of these folds, and then the performance of the model is evaluated on the remaining test set. For the DPI task, the benchmark dataset is divided into training, validation, and test sets at a ratio of 8:1:1. The model is trained on the training set, the validation set is used to verify the performance of the model in the iterative process, and finally the performance is evaluated on the test set. In addition, the choice of hyperparameters is based on previous experiments and experience. Detailed information on the hyperparameter settings can be found in Supplementary Table S4.

### 3.4 DTA prediction task

The experimental results are shown in Table 2. On the Davis dataset, HeteroDTA outperforms GSAML-DTA in MSE, CI, and $r_{m}^{2}$ by 8.46%, 1.00%, and 2.92%, respectively. Taking GraphDTA as an example, HeteroDTA not only improves the MSE index by about 19.65% but also improves the CI index by about 1.34%. On the KIBA dataset, HeteroDTA is improved by 9.09%, 0.78%, and 1.13% compared with GSAML-DTA. For the superiority of our model, there are three main reasons. Firstly, the multi-view compound feature extraction module can effectively leverage the chemical group information within a compound to comprehensively extract compound features from atom-level and group-level perspectives. Secondly, the integration of GEM and ESM-1b pre-trained models into HeteroDTA further enhances its representation learning ability, enabling the model to extract more comprehensive feature embedding representations and alleviating the problem of compound and protein data scarcity. Third, our proposed context-aware nonlinear feature fusion module outperforms the fusion methods in the baseline method. In summary, the experimental results demonstrate the effectiveness and superiority of our approach across various evaluation metrics compared to other methods.

**Table 2.** Comparison of model performance on two DTA benchmark datasets.

Method	Davis			KIBA		
	MSE (std)↓	CI (std)↑	$r_{m}^{2}$ (std)↑	MSE (std)↓	CI (std)↑	$r_{m}^{2}$ (std)↑
SimBoost	0.282 (–)	0.872 (±0.002)	0.644 (±0.006)	0.222 (–)	0.836 (±0.001)	0.629 (±0.007)
DeepDTA	0.261 (–)	0.878 (±0.004)	0.630 (±0.017)	0.194 (–)	0.863 (±0.002)	0.673 (±0.009)
DeepCDA	0.248 (–)	0.891 (±0.003)	0.649 (±0.009)	0.176 (–)	0.889 (±0.002)	0.682 (±0.008)
GraphDTA	0.229 (–)	0.893 (±0.001)	–	0.139 (–)	0.891 (±0.002)	–
WGNN-DTA	0.208 (–)	0.903 (–)	0.691 (–)	0.130 (–)	0.898 (–)	0.791 (–)
GSAML-DTA	0.201 (–)	0.896 (±0.001)	0.718 (±0.004)	0.132 (–)	0.900 (±0.004)	0.800 (±0.004)
HeteroDTA	**0.184** (±0.001)	**0.905** (±0.003)	**0.739** (±0.004)	**0.120** (±0.002)	**0.907** (±0.002)	**0.809** (±0.003)

Bold font indicates superior performance on evaluation metrics.

### 3.5 Compound–protein interaction prediction task

To fully evaluate the superiority of HeteroDTA in DPI tasks, we use the same data processing as the baseline model. Firstly, we make three sub-datasets of Human and *C.elegans* datasets based on different positive-to-negative sample ratios, 1:1, 1:3, and 1:5 respectively. We choose GraphDTA and WGNN-DTA as the comparison methods for the experiments. Supplementary Figure S3 shows that HeteroDTA exhibits excellent performance under different positive-to-negative sample ratios. Specifically, on the Human dataset, HeteroDTA shows excellent prediction performance with an AUC of 0.984, precision of 0.96, recall of 0.913, and F1-score of 0.936 at a positive-to-negative sample ratio of 1:1. As the number of negative samples increases, the prediction performances of the three models decrease. However, HeteroDTA maintains higher AUC values and F1-scores with positive-to-negative sample ratios of 1:3 and 1:5, suggesting that HeteroDTA is more adaptable to unbalanced data. On the *C.elegans* dataset, HeteroDTA also shows a significant advantage in dealing with unbalanced data compared to other methods. For example, with a 1:5 positive-to-negative sample ratio, HeteroDTA outperforms WGNN-DTA by about 2.1% in AUC value, and improves by about 18.5%, 3.4%, and 3.5% in Precision, Recall, and F1-score, respectively. In summary, HeteroDTA exhibits excellent predictive performance in the DPI task even in the presence of unbalanced data.

### 3.6 Ablation study

We evaluate the performance of each proposed core innovative element. Model-1 is chosen as the baseline method, which mainly relies on atom–bond graphs and residue contact graphs. Then, we improve the baseline method by adding the pharmacophore graph, the pre-trained model, and the feature fusion module, corresponding to Model-2, Model-3, and Model-4, respectively. The results of the baseline method and new models with different configurations are in Table 3. The results show that Model-2 improves the performance of the baseline method because the multi-view compound feature extraction model can extract key features from functional substructures and learn structural features comprehensively. According to the results obtained by Model-3, the pre-trained model generates more comprehensive and accurate feature embeddings by unsupervised learning on a large amount of unlabeled data, which is essential for capturing key features of proteins and compounds. The performance of Model-4 is further improved by combining the above three innovative elements. Specifically, on the Davis dataset, Model-4 achieves MSE and CI of 0.184 and 0.905, respectively, much better than the baseline method. This improvement is attributed to its context-aware nonlinear feature fusion mechanism, which enables the model to learn more detailed interaction patterns. Thus, the importance of feature fusion in deep neural networks is further validated, and more sophisticated feature fusion attention mechanisms may lead to better performance. In conclusion, the combination of all proposed innovation components achieves the best results compared to the baseline approach and other configurations, which validates the superiority of the different innovation elements.

**Table 3.** The ablation study results obtained on the Davis dataset.

Model	Pharmacophore	Pre-trained model	Feature fusion	MSE (std)↓	CI (std)↑	Spearman (std)↑
Model-1	–	–	–	0.206 (±0.002)	0.897 (±0.004)	0.862 (±0.003)
Model-2	✓	–	–	0.203 (±0.001)	0.901 (±0.003)	0.864 (±0.005)
Model-3	✓	✓	–	0.192 (±0.002)	0.903 (±0.003)	0.872 (±0.003)
Model-4 (HeteroDTA)	✓	✓	✓	**0.184** (±0.001)	**0.905** (±0.003)	**0.878** (±0.004)

Bold font indicates superior performance on evaluation metrics.

### 3.7 Generalization ability

**Figure 4.**
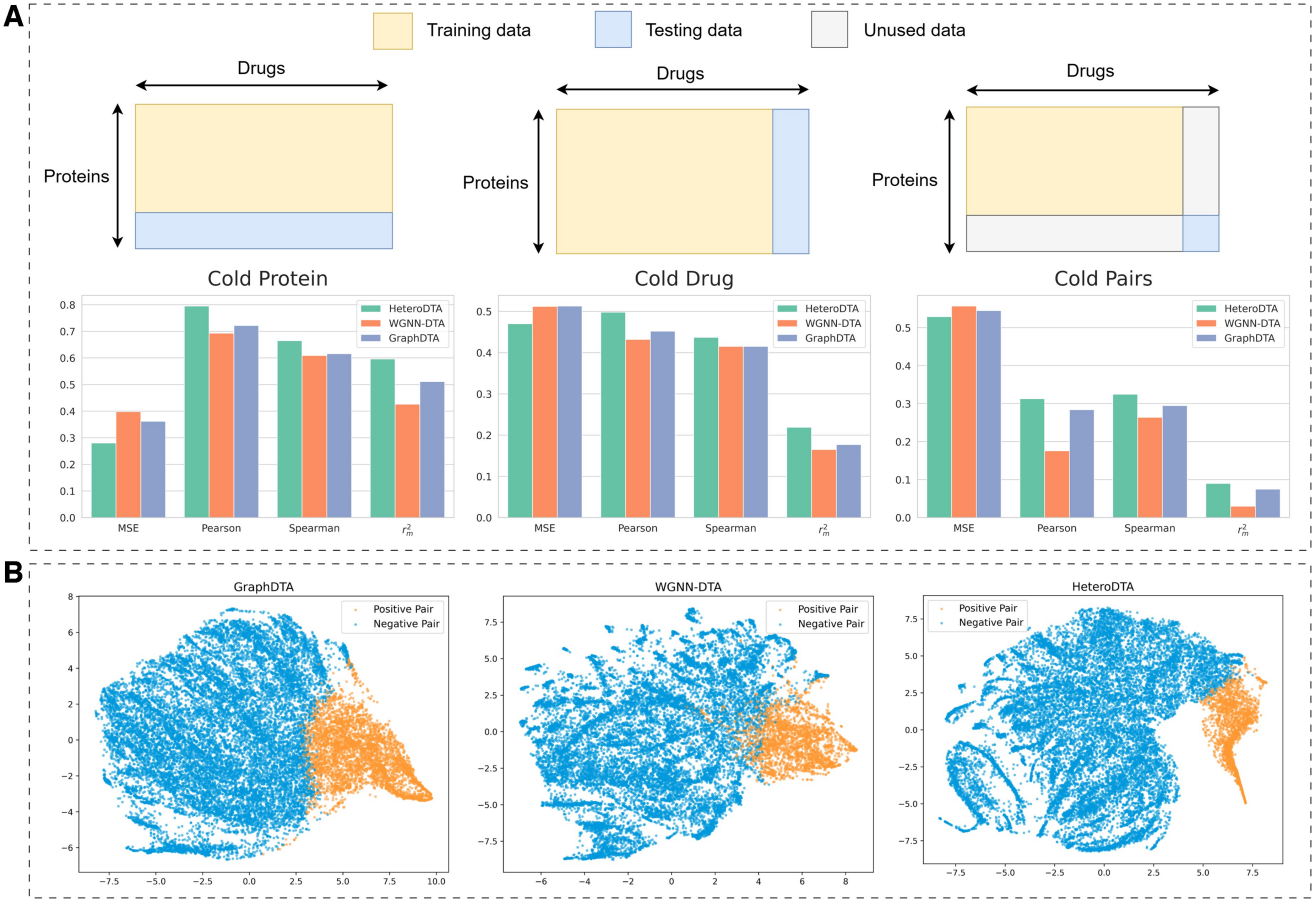
(A) Performance comparison of HeteroDTA with WGNN-DTA and GraphDTA on the Davis dataset under three cold-start scenarios. (B) Visualization of the drug–target pair representations using t-SNE on the KIBA test set.

In virtual screening, the cold-start scenario refers to the problem of dealing with unknown compounds or targets that do not appear in the training set, which is crucial to measure the generalization ability of the model. Three different cold-start scenarios are constructed on the Davis dataset to evaluate the performance of the model under unknown conditions: a cold-start drug scenario, a cold-start protein scenario, and a cold-start drug–target pair scenario. In the cold-start drug scenario, the drugs in the test set are not present in the training set, but all proteins are present in both sets. In the cold-start protein scenario, the proteins in the test set are not present in the training set, but all drugs are present in both sets. Finally, the cold-start drug–target pair scenario involves the prediction of interactions between unknown drugs and proteins, which is used to evaluate the generalization ability of the model in completely unseen scenarios. As shown in Figure 4, HeteroDTA shows significant advantages in all metrics under three cold-start scenarios. First, for the cold-start drug scenario, HeteroDTA demonstrates significant improvements compared to WGNN-DTA, enhancing MSE by 7.8%, Pearson by 15.7%, and $r_{m}^{2}$ by 32.7%. Furthermore, HeteroDTA also shows superior performance compared to other methods in the cold-start protein scenario and the more challenging cold-start drug–target pair scenario. The benefits of HeteroDTA can be attributed to the following: First, HeteroDTA constructs pharmacophore graphs of compounds, which decompose complex chemical structures into smaller group units to obtain a comprehensive understanding of the structural composition of compounds. Second, the pre-trained model introduces the rich knowledge learned in large drug or protein unlabeled databases, which effectively mitigates the scarcity of compounds and proteins in the DTA dataset. Finally, unlike WGNN-DTA and GraphDTA, which directly contact compound and protein features, HeteroDTA proposes a context-aware nonlinear feature fusion module that can learn the interaction pattern between compounds and proteins.

### 3.8 Representation power

Deep learning models predict the binding affinity of drugs to their targets by projecting the embedding representations of drug–target pairs into the underlying representation space and then using these representations to predict the binding affinity. Therefore, there is a correlation between the embedding representations of drug–target pairs and the prediction performance. This section aims to evaluate the representation learning ability of HeteroDTA in the DTA prediction task. Two benchmark datasets, Davis and KIBA, are used for the experiments. All models are pre-trained on the benchmark dataset, and then embedding representations of drug–target pairs are extracted from the penultimate layer of the prediction module of each model. The representation learning ability is evaluated by comparing its clustering effect on drug–target pairs in the embedding space. According to the results in Table 4, HeteroDTA performs better in clustering than other models on both datasets. For example, on the Davis dataset, HeteroDTA outperformed WGNN-DTA by 1.8%, 56.2%, and 22.2% on SC, CHI, and DBI metrics, respectively. In addition, the embedding representation of the KIBA test set is also visualized using *t*-distributed random neighbor embedding projections (t-SNE) into a 2D map (see Figure 4). The results show that HeteroDTA can distinguish positive and negative samples more clearly than other methods in the embedding space. In summary, through qualitative and quantitative analyses, it is demonstrated that HeteroDTA has superior representation learning ability and can clearly distinguish different clusters in the embedding space.

**Table 4.** Clustering performance of different models.

Model	Davis			KIBA		
	SI↑	CHI↑	DBI↓	SC↑	CHI↑	DBI↓
GraphDTA	0.569	4582.287	0.876	0.448	14972.723	0.929
WGNN-DTA	0.710	6537.96	0.748	0.491	7346.735	1.347
HeteroDTA	**0.723**	**10212.413**	**0.583**	**0.669**	**26717.827**	**0.641**

Bold font indicates superior performance on evaluation metrics.

### 3.9 Case study

Recent studies have identified the major protease (Mpro) as a central target of SARS-CoV-2. A total of about 300 antiviral drugs are collected, of which 10 have been validated in the literature for the treatment of SARS-CoV-2 (Supplementary Figure S4). To efficiently screen active compounds, three deep learning-based methods are used: HeteroDTA, GraphDTA, and WGNN-DTA. These methods are pre-trained on the KIBA dataset and then used to screen the compound library. The detailed workflow is shown in Supplementary Figure S5. The screening results are shown in Supplementary Table S5, in addition, the screening results are visualized using heatmap (Supplementary Figure S6). HeteroDTA showed the best performance in terms of enrichment rate. Specifically, six potentially active compounds have been identified among the top 10 compounds screened by HeteroDTA. For example, Simeprevir is ranked 1st, while it is ranked 264th and 218th in WGNN-DTA and GraphDTA, respectively. Similar trends are also observed for other active compounds, such as Hydroxychloroquine and Chloroquine. To further explore the relationship between the active compounds, the entire library of compounds is projected onto the t-SNE plot (Supplementary Figure S7). The results show that the active compounds are far apart from each other and there is no aggregation or overlap. In addition, Tanimoto similarity is calculated between the top 10 compounds, and the results showed that HeteroDTA screened these active compounds to the top position, not because of the strong structural similarity between them, but because HeteroDTA considered the structural information of the compounds and the targets for affinity prediction at the interaction level. For example, the Tanimoto similarity between Simeprevir (Rank1) and Hydroxychloroquine (Rank3) is 0.26 (below 0.5).

To further visualize the internal mechanism of HeteroDTA in affinity prediction, we use gradient-weighted class activation mapping (Grad-AAM) to improve the interpretation ability of the deep learning-based network model by visualizing the graph structure regions with the larger contribution to the prediction in the form of the heat map. Specifically, three antiviral compounds are selected as examples to illustrate the contribution of individual atoms in these compounds to their interaction with Mpro. The binding modes of the three compounds to Mpro are investigated through molecular docking by Hosseini *et al.* (2021). As shown in Supplementary Figure S8, the atomic contribution captured by HeteroDTA is to some extent consistent with the results of molecular docking. Among them, Chloroquine shows the highest degree of coincidence, which verifies the ability of HeteroDTA to capture the key binding mode. In the case of Lopinavir and Ritonavir, HeteroDTA identifies several active structures that can form hydrogen bonds, highlighting its efficacy in identifying potential key interaction points. Finally, it is worth mentioning that the screening process of HeteroDTA is very efficient and takes no more than 1 s except for data preprocessing. This combination of efficiency and accuracy demonstrates the advantages of HeteroDTA in identifying promising drug candidates and validates its potential for application in real-world drug discovery scenarios.

## 4 Conclusion

In this study, a novel deep learning-based small molecule drug screening model called HeteroDTA is being proposed. The main contributions of this model are as follows. First, a multi-view compound feature extraction module incorporating pharmacophore structures is introduced, which captures the features of compounds more comprehensively by integrating the compound information from different views. Second, the generalization ability is enhanced by incorporating advanced pre-trained models that offer a more comprehensive representation of compounds and proteins. This enhancement enables the model to better adapt to unseen data and improve performance in different tasks. Third, a context-aware nonlinear fusion mechanism is designed to learn the interaction patterns between compounds and proteins to better capture the complex relationships between them. Numerous experiments show that HeteroDTA achieves better performance compared to existing methods on various datasets, especially in complex cold-start scenarios. In addition, an embedding representation clustering experiment on drug–target pairs is conducted to demonstrate the excellent performance of HeteroDTA in representation learning, which can clearly distinguish between positive and negative samples. In the case study, efficient virtual screening of compound libraries against the therapeutic targets of SARS-CoV-2 is performed to validate the ability of HeteroDTA in a real-world drug discovery scenario. In the future, we will further explore other promising strategies, including but not limited to transfer learning, data augmentation, and generative adversarial networks, to improve the model performance when dealing with limited data in bioinformatics. Notably, HeteroDTA is expected to seamlessly integrate with traditional drug design methods to provide a more efficient solution for large-scale virtual screening, leading to more advances in drug development.

## Supplementary Material

btae240_Supplementary_Data

## Data Availability

Our study used open-access datasets. These dataset and source code of HeteroDTA are available in the github repository mentioned above.
