# Supplementary material for: Enhancing generalizability and performance in drug–target interaction identification by integrating pharmacophore and pre-trained models
Source: Bioinformatics. 2024 Jun 28;40(Suppl 1):i539–47. doi: 10.1093/bioinformatics/btae240 (PMC11211825; doi:10.1093/bioinformatics/btae240)
Supplement: btae240_Supplementary_Data [file btae240_supplementary_data.pdf]

# Supplementary Material

# Enhancing Generalizability and Performance in Drug-Target Binding Affinity Prediction by Integrating Pharmacophore Modeling and Pre-trained Neural Networks

## Section S1

In that section, all the supplementary tables mentioned in the paper will be listed here.

**Supplementary Table S1.** Physical and chemical properties of pharmacophore graph nodes.

| Property | Dimension |
|---|---|
| Atomic types contained in the node | 44 |
| Number of atoms in the node | 11 |
| Number of edges connecting to other nodes | 11 |
| Total number of hydrogen atoms connected by node | 12 |
| Implicit valence of node | 12 |
| The node is a simple ring | 1 |
| The node is chemically bonded or not | 1 |

**Supplementary Table S2.** Edge properties of compounds.

| Property | Dimension |
|---|---|
| Single bond | 1 |
| Double bond | 1 |
| Triple bond | 1 |
| Aromatic bond | 1 |
| Conjugated bond | 1 |

| | |
|---|---|
| Bond in ring | 1 |
| Bond stereochemistry | 4 |

**Supplementary Table S3.** Physicochemical properties of residues.

| Property | Dimension |
|---|---|
| One-hot encoding of the residue type | 21 |
| Residue weight | 1 |
| Whether the residue is aromatic | 1 |
| Whether the residue is aliphatic | 1 |
| Whether the residue is acidic charged | 1 |
| Whether the residue is basic charged | 1 |
| Whether the residue is polar neutral | 1 |
| The negative of the logarithm of the dissociation constant for the –COOH group | 1 |
| The negative of the logarithm of the dissociation constant for the –NH3 group | 1 |
| The negative of the logarithm of the dissociation constant for any other group | 1 |
| The pH at the isoelectric point | 1 |
| Hydrophobicity of residue (pH=2) | 1 |
| Hydrophobicity of residue (pH=7) | 1 |

**Supplementary Table S4.** The hyperparameters of HeteroDTA.

| Hyperparameters | Setting |
|---|---|
| Epoch | 2000 |
| Optimizer | Adam |
| Learning rate | 0.0005 |
| Batch size | 512 |
| Activation function | ReLU |
| Dropout rate | 0.2 |
| GNN layers | 3 |
| Heads of GAT | 1 |

| | |
|---|---|
| Dropout rate | 0.2 |
| GNN pooling layer | Global max pooling |
| Hidden layers for ESM-1b | 2 |
| Embedding dim of GEM | 32 |

**Supplementary Table S5.** Ranking results of virtual screening for SARS-CoV-2 based on deep learning.

| Compound | DrugBankID | HeteroDTA | WGNN-DTA | GraphDTA |
|---|---|---|---|---|
| Simeprevir | DB06290 | 1 | 264 | 218 |
| Hydroxychloroquine | DB01611 | 3 | 55 | 139 |
| Chloroquine | DB00608 | 4 | 28 | 93 |
| Methisazone | DB13641 | 6 | 43 | 72 |
| Cobicistat | DB09065 | 7 | 31 | 175 |
| Glecaprevir | DB13879 | 9 | 251 | 220 |
| Raltegravir | DB06817 | 31 | 93 | 8 |
| Ritonavir | DB00503 | 52 | 19 | 281 |
| Darunavir | DB01264 | 89 | 12 | 250 |
| Tipranavir | DB00932 | 129 | 77 | 88 |

## Section S2

In this section, all the supplementary figures mentioned in the paper will be listed here.

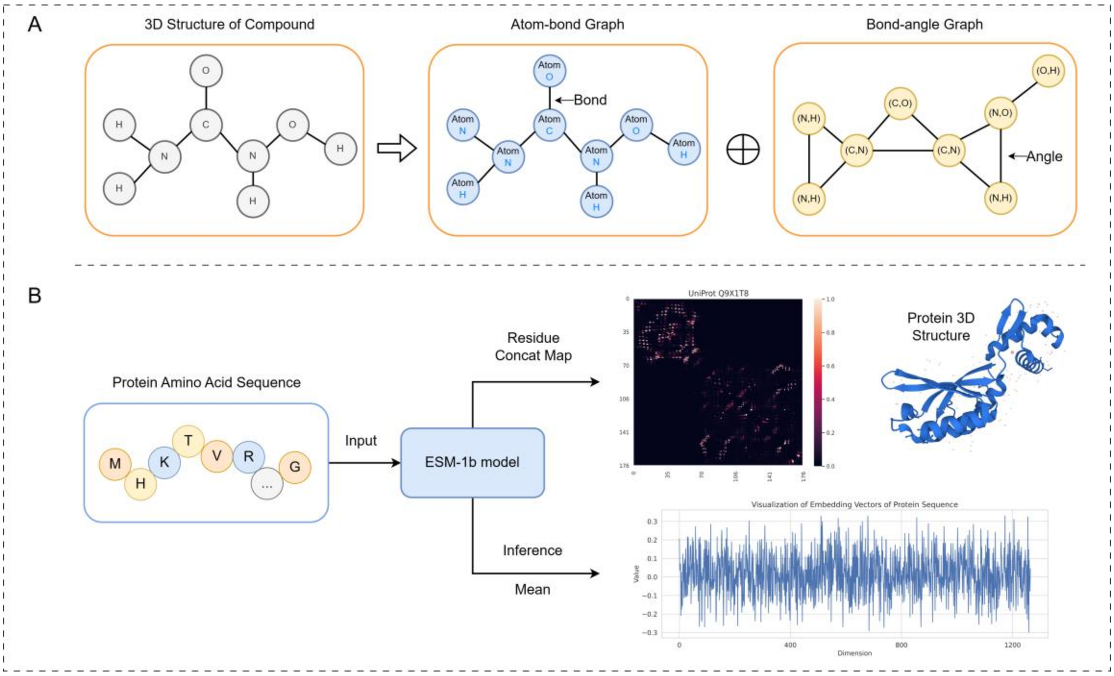


**Supplementary Fig. S1.** (A) The process of constructing an atom-bond graph and bond-angle graph of the compound. (B) Workflow for construction of residue concat graph and protein sequence embedding vectors using protein amino acid sequences.

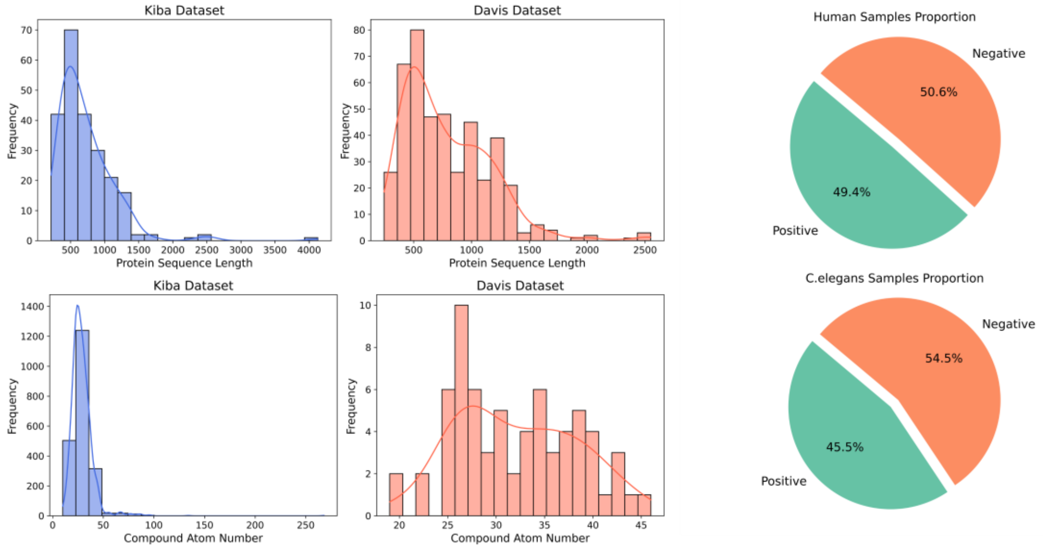


**Supplementary Fig. S2.** Statistics of all benchmark datasets. The KIBA dataset contains 2111 compounds and 229 proteins, totaling 118,254 samples. The Davis dataset contains 68 drugs and 442 targets, totaling 30,056 samples. The human dataset contains 2573 compounds, 1764 targets, and 5837 positive and 2951 negative samples. The C.elegans dataset contains 1733 compounds, 1735 targets, 3181 positive samples, and 3814 negative samples.

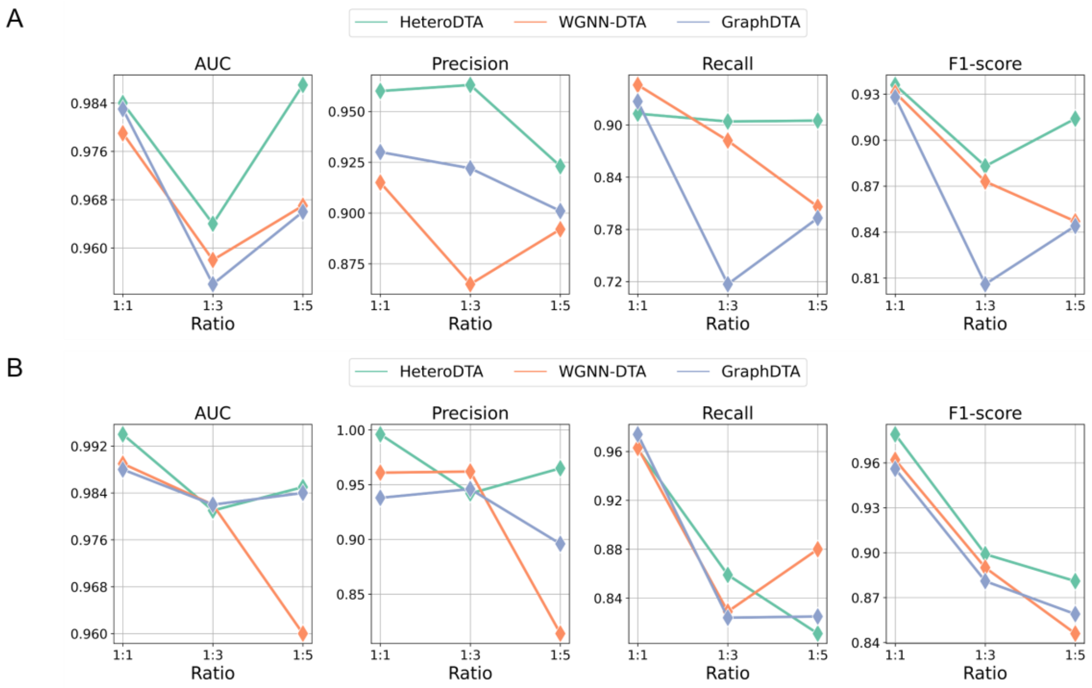


**Supplementary Fig. S3.** Comparison of model performance on Human dataset (A) and C.elegans dataset (B).

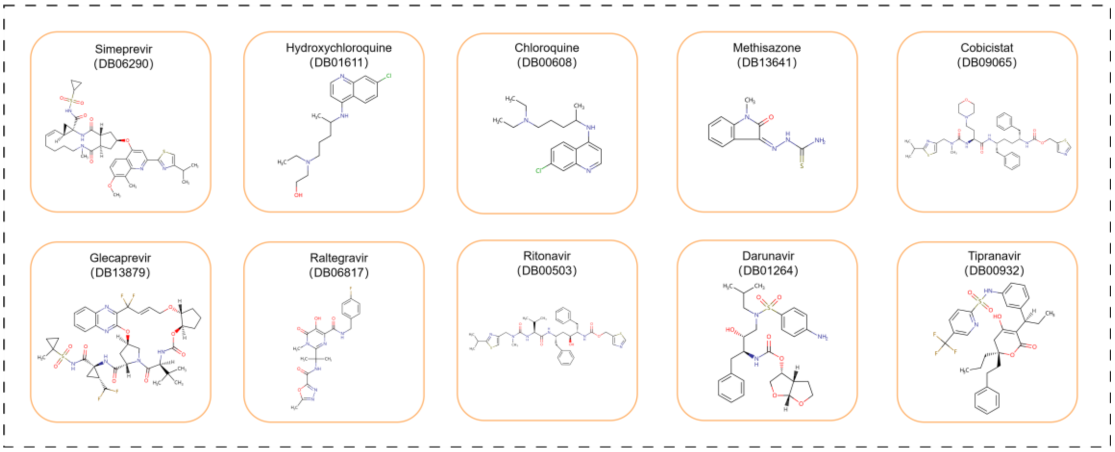


**Supplementary Fig. S4.** Structure and Drug Bank ID of 10 Active Compounds.

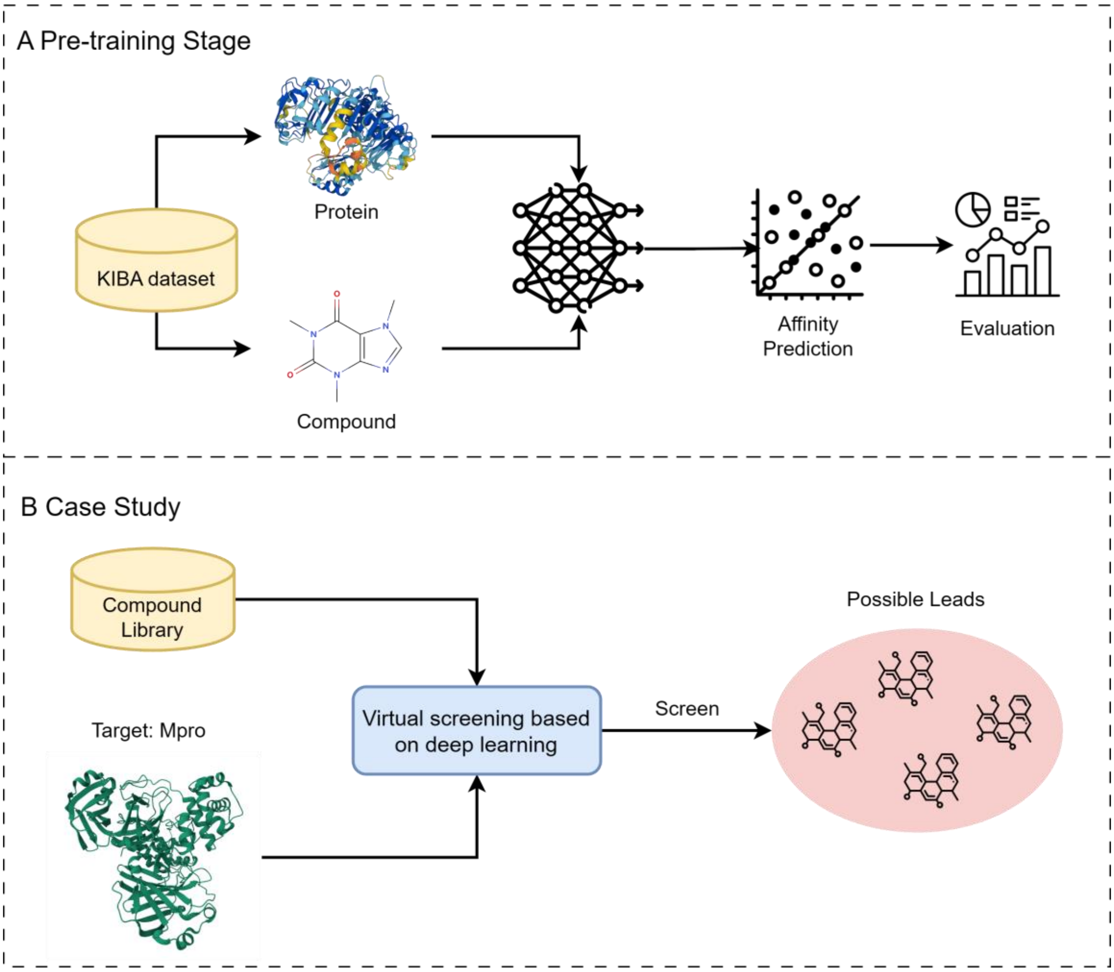


**Supplementary Fig. S5.** The workflow of virtual screening based on deep learning. (A) The deep learning model is initially pre-trained on the KIBA dataset, followed by an assessment of its performance. (B) Chemical library and disease target (Mpro) are collected, and then the pre-trained model is used for virtual screening to identify promising lead compounds.

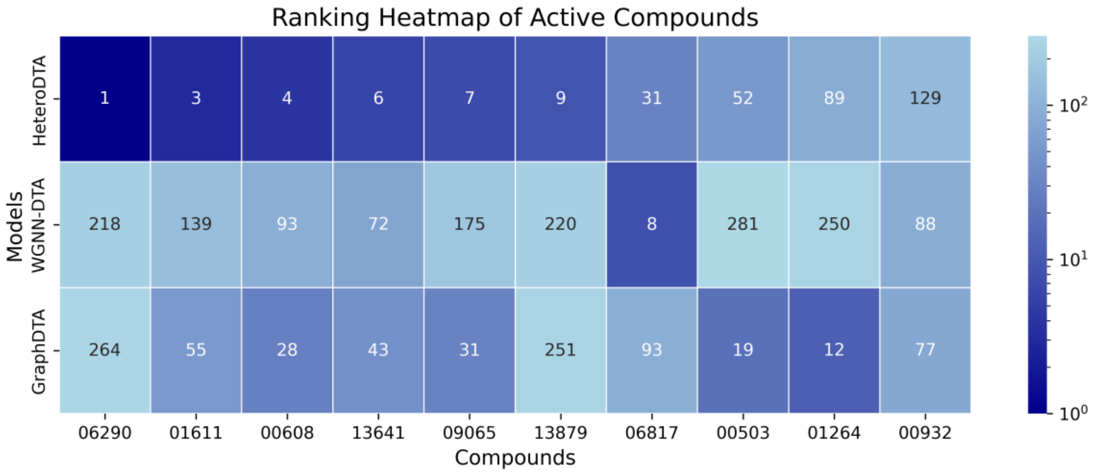


**Supplementary Fig. S6.** Heatmap of ranking for active compounds. The horizontal axis represents the Drug Bank ID, while the vertical axis represents the three deep-

learning models used in the case study. The color intensity indicates the ranking level.

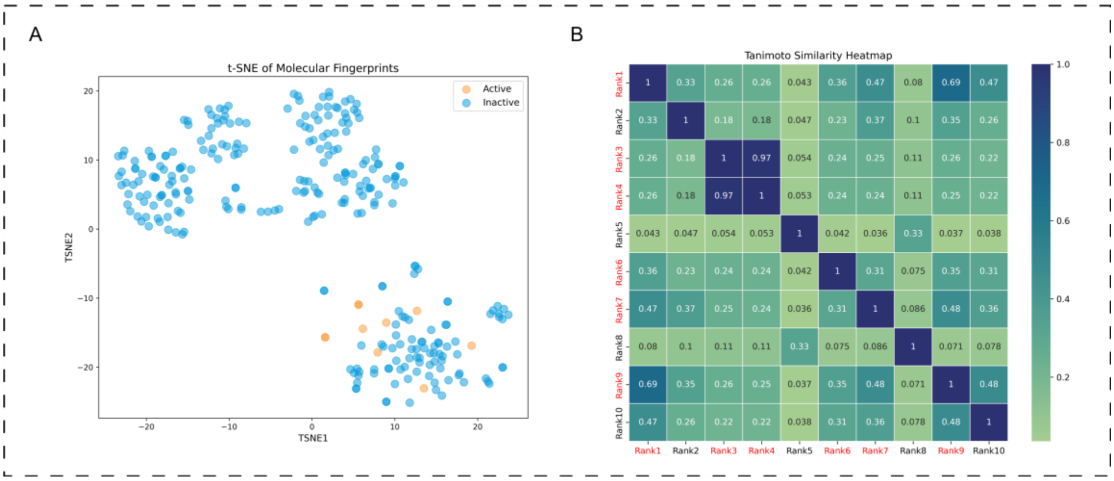


**Supplementary Fig. S7.** (A) t-SNE plot of the molecular fingerprint of the compound library. Yellow dots indicate active compounds and blue dots indicate inactive compounds. (B) Tanimoto similarity heatmap of the top ten compounds ranked by HeteroDTA. The red label indicates the active compound.

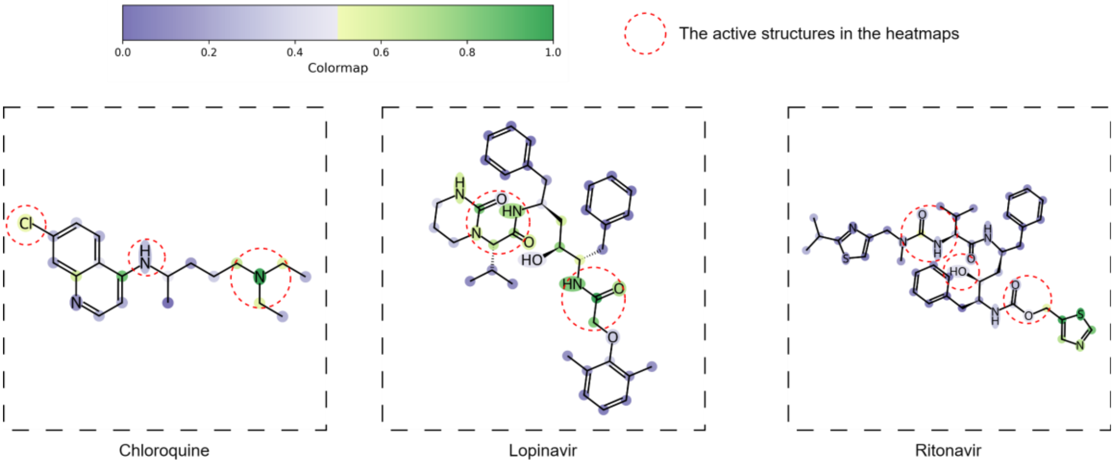


**Supplementary Fig. S8.** Interpretability diagram of the HeteroDTA model. Three antiviral compounds (Chloroquine, Lopinavir, and Ritonavir) are listed there, and the contribution of individual atoms in the compound structure is visualized using heat maps to assist pharmaceutical chemists in finding and optimizing the active structure.
